# Molecular Mechanisms of Cadmium-Induced Toxicity and Its Modification

**DOI:** 10.3390/ijms26157515

**Published:** 2025-08-04

**Authors:** Jin-Yong Lee, Maki Tokumoto, Masahiko Satoh

**Affiliations:** Laboratory of Public Health, School of Pharmacy, Aichi Gakuin University, Kusumoto-cho 1-100, Chikusa-ku, Nagoya 464-8650, Japan

**Keywords:** cadmium, renal toxicity, transcript pathway, apoptosis, iron deficiency anemia

## Abstract

Cadmium (Cd) is a toxic environmental heavy metal that exerts harmful effects on multiple tissues, including the kidney, liver, lung, and bone, and is also associated with the development of anemia. However, the precise molecular mechanisms underlying Cd-induced toxicity remain incompletely understood. In this paper, we review the recent molecular mechanisms of Cd-induced toxicity and its modification, with a particular emphasis on our recent findings. Using a combination of DNA microarray analysis, protein–DNA binding assays, and siRNA-mediated gene silencing, we identified several transcription factors, YY1, FOXF1, ARNT, and MEF2A, as novel molecular targets of Cd. The downregulation of their downstream genes, including *UBE2D2*, *UBE2D4*, *BIRC3*, and *SLC2A4*, was directly associated with the expression of cytotoxicity. In addition, PPARδ plays a pivotal role in modulating cellular susceptibility to Cd-induced renal toxicity, potentially by regulating apoptosis-related signaling pathways. In addition to apoptosis pathways, Cd toxicity through ROS generation, ferroptosis and pyroptosis were summarized. Furthermore, it has been revealed that Cd suppresses the expression of iron transport-related genes in duodenal epithelial cells leading to impaired intestinal iron absorption as well as decreased hepatic iron levels. These findings provide a mechanistic basis for Cd-induced iron deficiency anemia, implicating disrupted iron homeostasis as a contributing factor.

## 1. Introduction

Cadmium (Cd) is a heavy metal (atomic weight 112.41, density 8.6 g/cm^2^) with a melting point of 320.9 °C and boiling point of 767 °C [1]. It was discovered in 1817 by the German mineralogist F. Strohmeyer [2]. In the periodic table, Cd is in group 12 (between zinc and mercury). Cd has been used in pigments, batteries, alloys, and electroplating, as well as in automobile parts, electronic devices, camera components, plastic stabilizers, and nuclear reactor control rods [3,4,5]. In nature, Cd coexists with zinc, copper, lead, and other metals, and is obtained as a byproduct during the mining and smelting of those metals [2,6,7]. Cd, along with arsenic, lead, mercury, and chromium, is a heavy metal lacking any known physiological function and is widely recognized as a toxic environmental contaminant [8,9,10,11,12,13]. Cd is a ubiquitous environmental contaminant found in soil, water, air, and food sources [14,15,16,17,18,19]. Cd enters the human body mainly through dietary intake, smoking, and occupational exposure. Its long biological half-life (>10 years) and accumulation in organs such as the liver, kidney, and pancreas reflect its relevance as a chronic public health hazard [20,21,22,23,24].

As an international standard for the safe intake of Cd, the Joint FAO/WHO Expert Committee on Food Additives (JECFA) has established a Provisional Tolerable Monthly Intake (PTMI) of 25 μg/kg body weight [25]. In addition, maximum levels for Cd in food, including rice, have been set by the Codex Alimentarius Commission (CAC) of FAO/WHO [26] (see Table 1 for reference values).

Cd exposure to humans has been associated with damage to tissues including the kidney, bone, lung, reproductive system, and cardiovascular system in cases of occupational exposure (e.g., smelters, battery factories) and environmental contamination via food and water [2,27,28,29,30,31,32,33]. In particular, chronic renal toxicity, characterized by proximal tubular dysfunction, is a major toxic outcome in the safety assessment of Cd [34,35].

In Japan, in addition to occupational Cd poisoning, chronic Cd renal toxicity has occurred in several polluted areas (Fuchu in Toyama, Ikuno in Hyogo, the Kakehashi River basin in Ishikawa, Kosaka in Akita, and Tsushima in Nagasaki) in the past [36]. In 1955, in the Jinzu River basin of Toyama, the prolonged oral intake of rice and drinking water contaminated with Cd led to an outbreak of Itai-itai disease, which was characterized by renal damage and osteomalacia, primarily in older women [1,37]. Itai-itai disease, a pollution-related illness, occurred after the discharge of Cd-containing wastewater from the zinc refinery upstream of the Jinzu River [36,37]. This led to the Cd contamination of drinking water, soil, and agricultural crops, particularly rice, in the downstream area, Fuchu.

In addition to Japan, Cd pollution has also been confirmed in river basins near zinc mines and zinc smelters in Thailand and South Korea in the past, causing health damage to residents in the downstream area [38,39,40]. Furthermore, EFSA (European Food Safety Authority) recommends reducing Cd exposure, as the estimated mean dietary intake among adults in the EU is close to or slightly exceeds the tolerable weekly intake [41].

Recently, Cd poisoning caused by exposure in industrial workplaces or environmental pollution has rarely been observed in Japan. However, trace amounts Cd are present in various foods, including rice, and greater than 40% of the Cd intake by the Japanese population comes from eating rice [36,42]. Thus, Cd is continuously ingested through the diet over a lifetime. Because of its extremely long biological half-life (15–30 years) and tendency to accumulate in the body, there is growing concern about the health effects of long-term and low-level exposure to Cd, particularly among the elderly.

Studies of the mechanisms of Cd toxicity have been carried out for many years by various research groups worldwide. However, the molecular mechanisms of Cd-induced toxicity remain almost unknown. In this article, we review the molecular mechanisms of Cd toxicity and protective factors against it, focusing on the findings from our research.

## 2. Identification of Target Genes in Cd Renal Toxicity

It has been reported that toxic chemicals trigger cell death through several target genes [43,44,45]. To identify the target genes involved in Cd renal toxicity, we performed a genome-wide DNA microarray analysis of Cd-responsive genes in rat renal proximal tubular (NRK-52E) cells [46]. The exposure of NRK-52E cells to Cd upregulated 73 genes (>2-fold), including stress response genes such as metallothionein 1 (*Mt1*) and heme oxygenase 1 (*Hmox1*). In addition, 42 genes were downregulated to less than half their control expression; among these was *Ube2d4* (ubiquitin-conjugating enzyme E2 D4), which encodes a ubiquitin-conjugating enzyme of the Ube2d family that functions in the ubiquitin-proteasome system for selective protein degradation [46]. Furthermore, to the NRK-52E cell analysis, we have identified numerous Cd-responsive genes in other models, including human proximal tubular (HK-2) cells [47], mouse liver [48], mouse kidney [49], and mouse fetal liver [50].

Prior to the onset of overt cytotoxicity in HK-2 cells, Cd exposure upregulated the expression of 30 genes more than 2.0-fold, including seven genes encoding heat shock proteins. Concurrently, Cd downregulated the expression of 21 genes to less than 50% of control levels, including transcription-related genes such as *AP2B1* (adaptor-related protein complex 2 subunit beta 1), *HOXA7* (homeobox A7), *HOXA9*, and *TCEB2* (transcription elongation factor B polypeptide 2). Genes involved in defending against Cd toxicity including the zinc transporter *ZIP1* (*SLC39A1*) and the heat shock proteins *HSPH1* [heat shock protein family H (Hsp110) member 1] and *HSPA8* [heat shock protein family A (Hsp70) member 8] were among those altered by Cd exposure in HK-2 cells [51,52].

Hepatic transcriptional responses were assessed in mice administered 50 ppm Cd via drinking water for 30 days using DNA microarray analysis [48]. Exposure to Cd resulted in the upregulation of 2 genes and downregulation of 15 genes in the liver, suggesting early molecular alterations might precede the onset of hepatotoxic damage [48]. Mice were fed a control diet or a diet containing 300 ppm Cd for 12 months [49]. Compared with control animals, the hepatic expression levels of 32 genes, including *Hmox1* and *Mt2*, were upregulated more than 2.0-fold, whereas 113 genes, including those involved in transport and ubiquitination, were downregulated less than 0.5-fold [49]. These findings indicate substantial transcriptional reprogramming in response to chronic dietary Cd exposure.

Pregnant mice were administered Cd at a dose of 5 mg/kg, and the fetal liver was collected for transcriptomic analysis using DNA microarrays [50]. Cd exposure during gestation resulted in the upregulation of 1669 genes (≥2.0-fold) and the downregulation of 194 genes (≤0.5-fold) in fetal liver. Differentially expressed genes were classified into functional categories including cell cycle and proliferation, apoptosis, cell growth and differentiation, cellular defense mechanisms, metabolism, transport, transcriptional regulation, signal transduction, metal homeostasis, and the ubiquitin–proteasome system [50]. These findings provide important insights into the molecular mechanisms of transcription pathways underlying fetal toxicity induced by prenatal Cd exposure.

## 3. Apoptosis-Related Cd Toxicity and Modification

### 3.1. p53-Dependent Apoptosis via the Suppression of UBE2D Family Genes

As described above, the DNA microarray analysis of NRK-52E cells revealed that Cd treatment reduced the expression of the *Ube2d4* gene by approximately half [46]. Moreover, it was found that the expressions of *Ube2d4* and other *Ube2d* family genes (*Ube2d1*, *Ube2d2*, and *Ube2d3*) were markedly suppressed prior to the onset of Cd-induced cytotoxicity [53]. This was accompanied by a notable upregulation of p53, an apoptosis-related protein [53]. It was confirmed that Cd does not lead to an increase in *p53* mRNA levels, nor does it exert a direct inhibitory effect on proteasome activity [53]. p53 is a short-lived protein that is normally targeted for ubiquitin-mediated degradation, a process in which Ube2d family ubiquitin-conjugating enzymes are involved [54,55]. Therefore, the Cd-induced overaccumulation of p53 protein is thought to result from the inhibition of p53 degradation related to the downregulation of Ube2d family genes. In addition, Cd treatment increased p53 phosphorylation and observed DNA fragmentation, indicating that Cd induces p53-dependent apoptosis [53]. Using HK-2 cells, it was demonstrated that the double knockdown of *UBE2D2* and *UBE2D4* resulted in elevated levels of intracellular p53 protein, which is critically involved in Cd-induced apoptosis, and is observed in cells treated with Cd [56].

The gene expressions of *Ube2d* family members and protein levels of p53 in the kidney of mice chronically exposed to Cd were also investigated. Mice fed a diet containing 300 ppm Cd for 6–12 months developed mild renal toxicity. In these Cd-exposed mice, renal *Ube2d1*, *Ube2d2*, and *Ube2d3* mRNA levels were significantly decreased, whereas p53 protein levels were significantly increased, consistent with our in vitro findings [53,56]. Furthermore, immunohistochemical analysis revealed that chronic Cd exposure caused a marked accumulation of p53, specifically in the renal proximal tubules, and apoptosis was detected at the same locations [53,56]. This demonstrated that Cd induces p53-dependent apoptosis specifically in proximal tubular cells in vivo [53,56].

**Figure 1:**
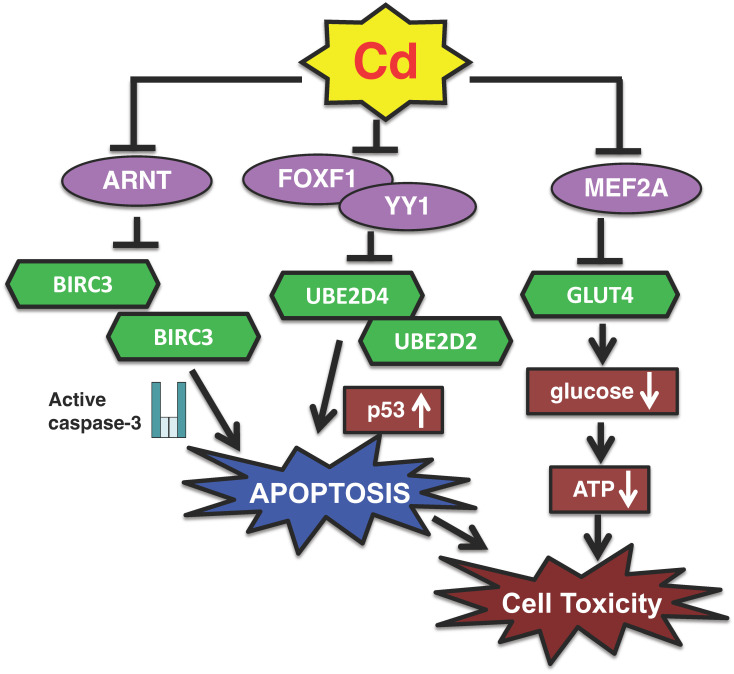
A new transcription pathway of Cd toxicity in proximal tubular cells. (1) Cd decreases the activities of YY1 and FOXF1 transcription factors and induces p53-dependent apoptosis by suppressing the gene expressions of *UBE2D2* and *UBE2D4*. (2) Cd inactivates the ARNT transcription factor and induces caspase-3-dependent apoptosis by suppressing the expression of *BIRC3*. (3) Cd suppresses the expression of *SLC2A4* coding GLUT4 protein via the inhibition of MEF2A transcriptional activity, resulting in reduced glucose uptake into cells, finally leading to cytotoxicity.

In summary, Cd specifically suppresses UBE2D (Ube2d) family gene expressions in cultured proximal tubular cells and in mouse kidney, leading to the overaccumulation of p53 and induction of p53-dependent apoptosis, thereby causing cytotoxicity (Figure 1). On the other hand, inorganic arsenic, but not inorganic mercury, also induced p53-dependent apoptotic pathways through the downregulation of gene expression of Ube2d family in proximal tubular cells [57].

### 3.2. Identification of Transcription Factors Rgulating UBE2D Family Gene Expression

Gene expression is regulated by transcription factors, and elucidating the mechanisms of transcript regulation is critical for understanding various biological phenomena [58,59]. The suppression of UBE2D family gene expression contributes to Cd-induced toxicity via apoptosis, implying that transcript regulation also underlies Cd toxicity; however, the specific transcription factors targeted by Cd remain largely unidentified. To determine which factors are involved in Cd toxicity, a protein/DNA binding array to identify transcription factors with DNA-binding activity altered by Cd was performed using NRK-52E cells [60]. Of 65 transcription factors examined, this analysis revealed that Cd treatment increased the activity of 6 transcription factors more than 2-fold and decreased the activity of 15 transcription factors less than 0.5-fold. In addition, using HK-2 cells, the protein/DNA binding assay was conducted to screen transcription factors affected by Cd [61]. The analysis revealed that exposure to Cd led to an increase in DNA-binding activity in 20 out of 345 transcription factors, whereas the activity of 28 transcription factors was downregulated [61].

YY1 (Yin-Yang 1) and FOXF1 (forkhead box F1) were among the transcription factors whose activities were decreased by Cd [56,60,61]. Moreover, the *UBE2D4* gene was shown to be regulated by FOXF1 [56]. In addition, *UBE2D2* gene expression was found to be regulated by YY1 [56]. The knockdown of *YY1* and *FOXF1* decreased cell viability as well as *UBE2D2* and *UBE2D4* activity, respectively [56]. Thus, YY1 and FOXF1 were identified as transcription factors that mediated the Cd-induced suppression of *UBE2D2* and *UBE2D4* gene expressions in HK-2 cells. Putative binding sites for YY1 and FOXF1 were identified in the upstream regulatory regions of *UBE2D2* and *UBE2D4*. However, YY1 specifically regulated the expression of *UBE2D2*, whereas FOXF1 was involved in the regulation of *UBE2D4* expression. These findings suggest that Cd may modulate gene expression via multiple pathways to influence the activity of specific transcription factors. Taken together, these results revealed that the transcription factors YY1 and FOXF1 are involved in the mechanism of Cd toxicity via the suppression of UBE2D family genes (Figure 1).

### 3.3. Apoptosis Induction via Inhibition of ARNT Transcriptional Activity

Based on the above findings, studies examined whether the silencing of individual transcription factors involved in Cd toxicity would alter cell viability. In HK-2 cells, the siRNA knockdown of *ARNT* (aryl hydrocarbon receptor nuclear translocator) significantly reduced cell viability, supporting the involvement of ARNT in Cd toxicity [61]. The mechanism by which Cd inactivates ARNT leading to cell toxicity was investigated. Although Cd treatment did not alter *ARNT* mRNA levels, it led to a reduction in ARNT protein levels in HK-2 cells. These findings suggest that ARNT is a potential target of Cd-induced renal toxicity at the post-transcriptional level. A microarray analysis of *ARNT*-knockdown HK-2 cells was performed to identify the downstream factors of ARNT. The results showed that the knockdown of *ARNT* led to the downregulation of 27 genes, each exhibiting expression levels reduced to less than 0.5-fold compared with control cells.

Among the genes whose activities were decreased by *ARNT* knockdown, *BIRC3* (baculoviral IAP repeat containing 3; also known as cIAP2) was investigated further. BIRC3 is a member of the inhibitor of apoptosis protein (IAP) family and suppresses apoptosis by inhibiting caspase-3 activation [62]. ARNT-mediated reduction in intracellular BIRC3 levels significantly decreased cell viability, induced apoptosis, and expressed Cd toxicity in HK-2 cells [61]. Moreover, it was confirmed that Cd inhibited transcriptional activity by reducing intracellular ARNT protein levels [61]. BIRC3 was reported to inhibit apoptosis by suppressing the activity of caspase-3, a key pro-apoptotic effector [62]. Our study revealed that knockdown of the BIRC3 gene activated caspase-3 in HK-2 cells, in a manner comparable with Cd treatment. Furthermore, we examined the kidney of mice with long-term Cd exposure and confirmed a significant decrease in *Birc3* mRNA levels [61]. We also found that Cd suppressed intracellular BIRC3 levels by reducing the intracellular levels of the ARNT transcription factor, thereby inhibiting its transcriptional activity, which in turn induced apoptosis. This represents a new apoptotic pathway of Cd toxicity involving ARNT and BIRC3 (Figure 1).

On the other hand, although methylmercury (MeHg) increased cleaved caspase-3 protein levels, mRNA levels of BIRC3 were not changed by MeHg in IMR-32 human neuroblastoma [61]. Inorganic mercury decreased mRNA levels of BIRC3 without cleaved caspase-3 change in HK-2 cells [61]. In mouse hepatic cells (AML-12 cells), arsenic increased the mRNA level of Birc3; however, the protein levels of cleaved caspase-3 were slightly increased by arsenic treatment [61]. Therefore, it is suggested that the activation of caspase-3 through suppression of BIRC3 gene expression by Cd treatment may mainly occur in proximal tubular cells.

### 3.4. Role of PPARδ as a Modification Factor of Cd Toxicity

To date, metallothionein and glutathione have been identified as key protective factors against Cd toxicity [1,34,63,64,65,66,67,68,69,70,71,72]; however, other defense mechanisms involved in Cd toxicity remain largely unknown. Recent our findings have demonstrated that Cd increased the activity of peroxisome proliferator-activated receptors (PPARs) in human and rat proximal tubular cells [46,56]. These results suggest that PPARs may be involved in susceptibility to Cd-induced renal toxicity. PPARs are ligand-activated transcription factors classified within the nuclear receptor superfamily and comprise three isoforms in humans: PPARα, PPARδ, and PPARγ [73,74,75,76,77]. Among the PPAR isoforms, only the knockdown of *PPARD* conferred significant resistance to Cd-induced toxicity in HK-2 cells and PPARδ transcriptional activity was attenuated in HK-2 cells subjected to Cd treatment or *PPARD* silencing [78]. To elucidate the effect of Cd treatment on *PPARD* expression in HK-2 cells, *PPARD* mRNA levels were measured following Cd exposure. Unexpectedly, Cd treatment significantly and dose-dependently increased *PPARD* mRNA expression [78]. These results suggest that the Cd-induced suppression of PPARδ transcriptional activity is unlikely to be attributable to reduced *PPARD* gene expression.

To identify genes regulated by PPARδ, DNA microarray analysis of HK-2 cells following transfection with *PPARD* siRNA was conducted. *PPARD* knockdown resulted in the upregulation of 53 genes more than 3-fold [78]. Several of the genes upregulated by *PPARD* knockdown, including *RYR2* (ryanodine receptor 2), *ITPK1* (inositol-tetrakisphosphate 1-kinase), *PALD1* (phosphatase domain containing paladin 1), *ZNF488* (zinc finger protein 488), *TFF2* (trefoil factor 2), *IL9R* (interleukin 9 receptor), *PANX2* (pannexin 2), *CPA4* (carboxypeptidase A4), *CCL19* (CC motif chemokine ligand 19), *FSIP1* (fibrous sheath interacting protein 1), and *MLXIPL* (MLX interacting protein like), are involved in apoptotic pathways. *PPARD* knockdown led to the downregulation of 39 genes, with expression levels reduced to ≤0.5-fold [78]. Of these downregulated genes, *LPAR3* (lysophosphatidic acid receptor 3), *GAL3ST1* (galactose-3-*O*-sulfotransferase 1), *PTPN11* (protein tyrosine phosphatase non-receptor type 11), and *RORA* (RAR related orphan receptor A) are involved in apoptotic pathways [78]. Microarray-based transcriptomic profiling indicated that *PPARD* knockdown altered the expressions of genes associated with apoptotic pathways. Further study demonstrated that Cd exposure increased the mRNA levels of *CPA4* and *FSIP1*, both of which were upregulated by *PPARD* knockdown [78]. Moreover, the upregulation of *CPA4* and *FSIP1* attenuated apoptotic signaling, suggesting their potential anti-apoptotic roles [79,80]. These results indicate that apoptosis may be involved in the enhanced resistance to Cd toxicity observed in *PPARD* knockdown cells.

Therefore, it was investigated whether the resistance to Cd toxicity observed in *PPARD* knockdown cells was associated with alterations in apoptotic processes. In control cells, Cd treatment significantly induced apoptosis and increased levels of cleaved caspase-3; however, *PPARD* knockdown attenuated the Cd-induced apoptosis and elevation of cleaved caspase-3 [78]. It is possible that *PPARD* knockdown altered the intracellular accumulation of Cd in HK-2 cells, which was increased in a dose-dependent manner with increasing Cd-treated concentrations [78]. However, *PPARD* knockdown had no effect on intracellular Cd levels [78].

**Figure 2:**
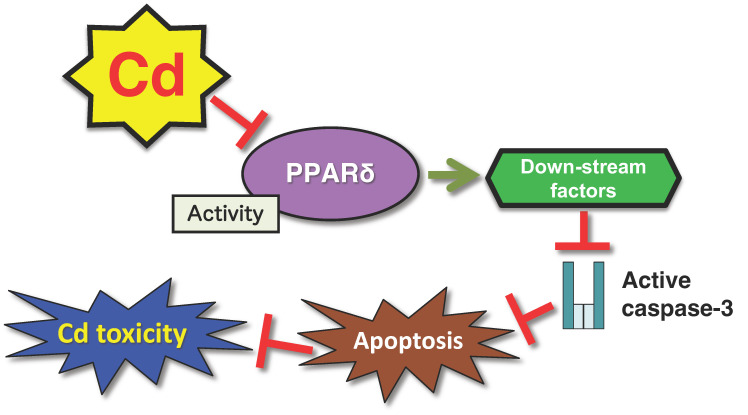
A defense mechanism against Cd toxicity mediated by the PPARδ transcription factor. Reduction in the activity of the PPARδ transcription factor suppresses Cd-induced apoptosis mediated by cleaved caspase-3.

This study provides strong evidence that the transcription factor PPARδ modulates cellular susceptibility to Cd toxicity. In HK-2 cells, PPARδ transcriptional activity was suppressed in response to Cd exposure. Of note, this Cd-induced reduction in PPARδ activity was associated with the attenuation of the apoptosis pathway, and *PPARD* knockdown conferred resistance to Cd toxicity (Figure 2). These findings suggest that the inhibition of PPARδ activity may be an adaptive cellular response to mitigate Cd-induced cytotoxicity. Furthermore, *PPARD* knockdown did not affect intracellular Cd concentrations, indicating that the observed resistance is not attributable to altered Cd uptake or excretion mechanisms in HK-2 cells [78].

### 3.5. Reactive Oxygen Species (ROS)-Related Apoptosis Involved in Cd Toxicity

Apoptosis is triggered through ROS generation from mitochondrial malfunction [81]. Occupational exposure to cadmium among workers in battery recycling and welding industries has been shown to result in cadmium accumulation in the lungs, leading to long-term toxic effects [82]. Recent findings have demonstrated that Cd induces cytotoxicity in human bronchial epithelial cells (BEAS-2B cells) through mitochondrial-mediated apoptosis and oxidative stress [83]. Exposure to Cd reduced mitochondrial membrane potential, while increasing intracellular ROS and apoptotic cell death [83]. Molecular analysis revealed downregulation of Bcl-2 (B-cell lymphoma 2) and upregulation of Bax (Bcl-2-associated X protein) and cleaved caspase-3, indicating activation of the intrinsic apoptotic pathway [83]. Additionally, Cd exposure enhanced the phosphorylation of JNK (c-Jun N-terminal kinase), ERK (extracellular signal-regulated kinase), and p38, suggesting involvement of the MAPK (mitogen-activated protein kinase) signaling pathways [83]. These results indicate that Cd induces apoptosis in bronchial cells by elevating ROS levels, activating MAPK signaling, and triggering mitochondria-dependent apoptotic mechanisms.

Cd exposure reduces the viability of A549 lung epithelial cells, accompanied by increased ROS, lipid peroxidation, and lactate dehydrogenase (LDH) leakage [84]. Antioxidant enzymes SOD (superoxide dismutase) and GSH-Px (glutathione peroxidase) were depleted, indicating oxidative stress [84]. Both intrinsic and extrinsic apoptotic pathways were activated, as evidenced by upregulation of TNF-α (tumor necrosis factor-α), caspase-8, and Bax, and downregulation of Bcl-2 [84]. These studies provide mechanistic insight into Cd-induced pulmonary cytotoxicity and contribute to understanding ROS-related apoptosis in Cd-related lung diseases.

### 3.6. Endoplasmic Reticulum (ER)-Mediated Apoptosis Involved in Cd Toxicity

Disruption of intracellular calcium (Ca^2+^) homeostasis has been shown to inhibit autophagy, promote oxidative stress, and subsequently induce apoptotic cell death [85]. Recent studies have demonstrated that activation of the calcium-sensing receptor (CaSR) mitigates Cd-induced cytotoxicity in mouse renal tubular epithelial cells (mRTECs) by restoring Ca^2+^ homeostasis in ER [86]. Cd exposure reduced SERCA2 (sarco/ER Ca^2+^-ATPase 2) expression and phosphorylation of its regulator, phospholamban (p-PLB), both in vitro and in vivo, contributing to ER stress and apoptosis [86]. Overexpression of SERCA2 effectively suppressed ER stress and cell death [86]. Notably, Cd promoted proteasomal degradation of SERCA2, as evidenced by rescue with the proteasome inhibitor MG132 [86]. These findings suggest that ER stress plays a central role in Cd toxicity through apoptosis, and that proteasomal regulation of SERCA2 stability may be suggested a therapeutic target to prevent Cd-induced nephrotoxicity.

Not only in renal tubular cells but also in MC3T3-E1 osteoblasts, Cd induces ER stress in a dose-dependent manner, leading to apoptosis [87]. Cd activated ER stress pathway through the PERK (protein kinase R (PKR)-like endoplasmic reticulum kinase)–eIF2α (eukaryotic translation initiation factor α)–ATF4 (activating transcription factor 4)–CHOP (C/EBP homologous protein) and suppressed the antioxidant response mediated by Nrf2 (Nuclear Respiratory Factor2)/NQO1 (NAD(P)H quinone dehydrogenase 1) [87]. Co-treatment with N-acetylcysteine (NAC) and 4-phenylbutyric acid (4-PBA) alleviated ER stress and attenuated Cd-induced apoptosis [87]. Furthermore, using the rats, Cd induced oxidative stress, renal tissue damage, and apoptosis by inhibiting the PERK signaling pathway [88]. These findings suggest that targeting oxidative stress and ER stress may provide protective strategies against Cd-induced tissue injuries.

## 4. Non-Apoptotic Cell Death Pathways Regulating Cd Toxicity

### 4.1. Suppression of GLUT4 via Inhibition of MEF2A, and Reduction in Intracellular Glucose

It was suggested that Cd may target MEF2A (myocyte enhancer factor 2) transcription factors in the pathway of renal toxicity [61]. MEF2A has been reported to regulate the transcription of *SLC2A4*, which encodes a glucose transporter 4 (GLUT4) [89,90]. We examined the effects of Cd exposure and *MEF2A* knockdown on *SLC2A4* expression and cellular glucose uptake in HK-2 cells [91]. The siRNA-mediated knockdown of *MEF2A* significantly decreased *SLC2A4* gene expression, and similarly, Cd exposure markedly reduced *SLC2A4* mRNA levels [91]. Cd also reduced GLUT4 protein levels in HK-2 cells [91]. Furthermore, the knockdown of *SLC2A4* itself caused a significant decrease in the viability of HK-2 cells [91]. These results suggest that the Cd-induced inhibition of MEF2A leads to the downregulation of GLUT4, which is involved in Cd cytotoxicity.

GLUT2 is highly expressed in the human kidney [92]; however, the knockdown of *SLC2A2* encoding GLUT2 did not affect the viability of HK-2 cells [91]. Notably, *MEF2A* knockdown specifically suppressed *SLC2A4* expression without reducing *SLC2A2* mRNA levels [91]. These findings suggest that GLUT4, but not GLUT2, is a key mediator in the Cd toxicity pathway, as a downstream factor of MEF2A inhibition.

Because GLUT4 is a glucose transporter, we measured intracellular glucose levels after Cd exposure and *SLC2A4* knockdown. Cd treatment and *SLC2A4* siRNA caused a significant decrease in intracellular glucose in HK-2 cells [91] suggesting that the Cd-induced suppression of GLUT4 leads to reduced glucose uptake into cells, contributing to cytotoxicity. Indeed, we found that HK-2 cells cultured in glucose-free medium, which lowers intracellular glucose levels, also reduced cell viability [91]. Intracellular glucose is metabolized to ATP [93]; therefore, changes in intracellular ATP levels were investigated in Cd-treated HK-2 cells and *SLC2A4*-knockdown cells. A significant reduction in ATP levels was observed following exposure to Cd and in *SLC2A4*-knockdown cells, indicating a potential link between *SLC2A4* expression and cellular energy metabolism. Taken together, our results indicate that Cd inhibits MEF2A, resulting in decreased GLUT4 levels and impaired glucose uptake, which in turn causes cytotoxicity (Figure 1).

Whether *Slc2a4* gene expression is suppressed following Cd accumulation in the mouse kidney has been investigated. Cd exposure for 6 or 12 months resulted in the accumulation of approximately 200 µg Cd/g kidney [53,56] and chronic Cd exposure led to a marked decrease in renal *Slc2a4* mRNA levels [91]. These findings suggest that, consistent with in vitro results, Cd-induced renal toxicity may involve impaired glucose transport in the kidney. This observation has important clinical implications for our understanding of the metabolic consequences of long-term Cd exposure.

### 4.2. Reactive Oxygen Species (ROS)-Necroptotic Cell Death Pathways Involved in Cd Toxicity

Cd is known to exert the cell toxicity through necrosis pathway [94,95,96,97,98]. Necroptosis has recently emerged as a noteworthy form of regulated cell death (RCD) contributing to cytotoxicity. Although necroptosis shares morphological features with necrosis, it is a form of RCD by specific intracellular factors, resembling apoptosis [99,100,101,102,103]. Recent studies have demonstrated that Cd induces necroptotic pathway in several tissues [104,105,106,107]. Cd exposure induces necroptotic cell death in Leydig cells, interstitial cells of the human testis, with significant involvement of the TNF-α/TNFR 1 (tumor necrosis factor receptor 1) signaling pathway and reactive oxygen species (ROS) generation. Co-treatment with Necrostatin-1 (Nec-1), a selective necroptosis inhibitor, effectively suppressed ROS accumulation and TNF-α/TNFR1-mediated necroptosis [107].

In swine small intestine, Cd activated the TNF-α/NF-κB (nuclear factor-kappa B) pathway and upregulated the levels of proinflammatory markers, such as HO-1 (heme oxygenase-1), IL-1β (interleukin 1β), iNOS (inducible nitric oxide synthase), COX2 (cyclooxygenase 2). These changes were alleviated by Nec-1 and N-acetylcysteine (NAC), highlighting the roles of necroptosis and ROS in Cd-induced intestinal toxicity [104]. These results suggest that necroptosis plays a pivotal role in Cd-induced dysfunction in several tissues.

### 4.3. Induction of Ferroptosis in Cd-Exposed Renal Cells

Ferroptosis is a recently identified form of RCD characterized by iron dependency and triggered by the accumulation of lipid peroxidation products and disturbances in cellular redox homeostasis [108,109,110,111]. A key contributor to this process is the Fenton reaction, wherein ferrous iron (Fe^2+^) reacts with hydrogen peroxide to generate hydroxyl radicals (·OH), a highly reactive species capable of inflicting severe oxidative damage [108,110,111,112]. Moreover, increasing evidence highlights glutathione peroxidase 4 (GPX4)—a selenium-containing enzyme—as a critical suppressor of ferroptosis, whose activity is closely linked to intracellular glutathione levels [113].

Recent evidence indicates that chronic low-dose Cd exposure induces ferroptosis and renal dysfunction. In mouse kidney and tubular epithelial cells, Cd exposure disrupted glutathione homeostasis and triggered ferroptotic cell death. It was further identified STEAP3-dependent lysosomal iron overload as a key driver of glutathione redox imbalance and ferroptosis [114]. These findings provide novel insights into the metabolic basis of Cd-induced nephrotoxicity, establishing STEAP3-mediated ferroptosis as a central mechanism.

Cd exposure was shown to downregulate mitochondrial SIRT3 (sirtuin 3), an NAD^+^-dependent deacetylase critical for mitochondrial protein regulation, in mouse kidney. This downregulation was associated with increased acetylation and decreased expression of mitochondrial GPX4. Particularly, Cd-induced GPX4 acetylation and ferroptotic cell death were further exacerbated in *Sirt3* knockout mice. Conversely, pretreatment with nicotinamide mononucleotide (NMN), a NAD^+^ precursor, attenuated mitochondrial oxidized lipid accumulation and suppressed GPX4 acetylation and ferroptosis in both HK-2 cells and Cd-exposed mouse kidney [115]. These findings suggest that SIRT3 downregulation promotes mitochondrial GPX4 acetylation, thereby enhancing susceptibility to Cd-induced ferroptosis in renal cells.

### 4.4. Inflammatory Pyroptosis in Cd Toxicity

Pyroptosis, also known as cell inflammatory necrosis, is a kind of RCD [99,116,117]. It causes cell swelling until the cell membrane ruptures, resulting in the release of cell contents and the activation of a strong inflammatory reaction [30]. Pyroptosis is an important natural immune response of the body, playing a vital role in fighting infection [30].

Recent studies using TM4 Sertoli cells have revealed that Cd significantly increases intracellular ROS, LDH, and interleukin-1β (IL-1β), while impairing mitochondrial function and promoting pyroptosis [118]. Mechanistically, Cd-induced pyroptosis is mediated by gasdermin D (GSDMD), pyroptotic effector protein, activation and inflammasome signaling, particularly through the ROS/NLRP3 (nucleotide-binding oligomerization domain-like receptor family pyrin domain-containing protein 3)/Caspase-1 axis [118]. These findings suggest that Cd induces GSDMD-mediated pyroptosis in Sertoli cells primarily through ROS-dependent inflammasome activation, offering potential targets for mitigating male reproductive toxicity caused by heavy metal exposure.

A recent mouse study demonstrated that intraperitoneal Cd exposure led to reduced testosterone levels, sperm count, and motility, along with increased LDH and IL-1β [119]. Histological and molecular analyses revealed oxidative stress, DNA damage, elevated ROS levels, and increased TUNEL-positive cells in the testis. Transcriptomic profiling indicated activation of inflammatory and chemokine pathways, particularly upregulation of AIM2 (absent in melanoma 2) and downregulation of NLRP3 [119]. Pyroptosis-related proteins, including GSDMD, GSDME, Caspase-1, ASC (apoptosis-associated speck-like protein containing a caspase recruitment domain), and IL-1β, were also elevated [119]. These findings suggest that Cd induces pyroptosis in testicular tissue primarily through AIM2-mediated inflammatory responses triggered by oxidative stress.

### 4.5. Lysosomal-Irregulated Autophagic Dysfunction in Cd Toxicity

Autophagy is a cellular process that eliminates damaged components via autophagosome–lysosome fusion to maintain homeostasis [120]. In the swine model, Cd exposure upregulated ER stress-related genes and altered calcium signaling in myocardium samples [121]. Particularly, autophagy-related genes, CAMKKII (calcium/calmodulin-dependent protein kinase kinase 2), ATG5 (Autophagy-related 5), LC3-II (light chain-3-II) were elevated, whereas mTOR (mammalian target of rapamycin) was suppressed. Notably, Cd impaired lysosomal function by downregulating V-ATPase and cathepsins (CTSB, CTSD) and promoting cathepsin leakage into the cytoplasm. Cd reduced autophagosome–lysosome colocalization, indicating autophagic flux blockage. These findings suggest that lysosomal dysfunction and impaired autophagy contribute to Cd-induced cardiotoxicity in swine.

Recent studies revealed that autophagy-related Cd toxicity is involved in glycolysis enhancing. In A549 human lung cancer cells, PHGDH (phosphoglycerate dehydrogenase) promoted Cd-induced autophagy, as well as Cd increased PHGDH expressions [122]. Furthermore, transfection with siATF4 inhibited Tm (ER stress inducer)-induced PHGDH expression. In summary, ATF4-mediated transcriptional regulation of PHGDH plays a pivotal role in Cd-induced autophagy initiated by ER stress. The ER stress–PHGDH–autophagy axis contributes to Cd-induced cell migration by promoting glycolytic activity. In human lung fibroblast cells, Cd-induced upregulation of mTOR was dependent on autophagy, while autophagy-driven cell growth and glycolysis required mTOR activation [123]. Autophagy activated mTOR-dependent glycolytic pathways, in which increased expression of GLUT1 (glucose transporter 1) facilitated glucose uptake and accelerated cell proliferation.

## 5. Molecular Mechanism of Cd-Induced Iron Deficiency

Chronic Cd toxicity can cause iron-deficiency anemia in addition to kidney dysfunction [124,125]. Studies in Japan and elsewhere have reported that Cd induced iron-deficiency anemia by reducing iron stores in the body [124,125]. It has been suggested that the intestinal absorption of Cd involves DMT1 (divalent metal transporter 1), an iron transporter [126,127]. However, the mechanism by which Cd causes iron-deficiency anemia remains largely unknown. Recent studies of this mechanism examined the effect of Cd on the expressions of iron transport-related genes in the intestine and on iron storage in the body, using animal and cell culture models [128,129].

Fujiwara et al. investigated the effect of a single oral dose of Cd on iron transporter expression in the duodenum of mice, as well as on serum iron levels. At 3 h after oral Cd administration, the duodenum showed significantly decreased mRNA levels of *Dmt1*, *Cybrd1* (cytochrome b reductase 1, *Dcytb*), *Fpn1* (ferroportin 1), and *Heph* (hephaestin) compared with controls [128]. Similarly, the mRNA level of *Hcp1* (heme carrier protein 1) was markedly reduced at 3 h post-Cd exposure, comparable with non-heme iron transporters [128]. Cd exposure also markedly suppressed Dmt1 and Fpn1 protein expressions in the duodenum [128]. Twenty-four h after Cd administration, the serum iron concentration was significantly lower in Cd-treated mice than in controls [128]. It was investigated the effect of Cd on iron transporter expression in a human intestinal cell model using Caco-2 cells (a human intestinal epithelial cell line). Exposure to 10 μM Cd significantly decreased the mRNA levels of *DMT1*, *CYBRD1*, *FPN1*, and *HEPH*, and reduced the protein expressions of DMT1 and FPN1 in Caco-2 cells [128]. These findings indicate that in Caco-2 cells, as in the mouse duodenum, Cd suppresses the expressions of *DMT1*, *CYBRD1*, *FPN1*, and *HEPH*. Thus, at the cellular level, Cd can directly downregulate iron transport molecules, potentially impairing iron uptake.

Tokumoto et al. investigated the effect of long-term exposure to Cd on iron transporter expression in the duodenum of mice [129]. Although the iron concentration in the liver was markedly decreased by Cd, the serum iron level was not markedly changed. Among the iron-transport-related genes in the proximal duodenum, the gene expressions of *Hcp1* and *Cybrd1* were significantly decreased by Cd [129]. Long-term exposure to Cd, especially, decreased the mRNA level of hepatic *Hamp* (hepcidin antimicrobial peptide), which degrades Fpn1 in the intestine [129]. The level of Fpn1 was maintained and this facilitated the transfer of iron from enterocytes to the blood.

Fujiwara et al. demonstrated that the serum iron concentration and total iron-binding capacity (TIBC) were significantly reduced 24 h after a single administration of Cd to mice [128]. In contrast, serum iron levels in mice subjected to long-term Cd exposure remained comparable with control levels, indicating that iron deficiency was not induced under these conditions [129]. However, hepatic iron accumulation was markedly reduced after 12 months of Cd exposure, and the unsaturated iron-binding capacity (UIBC) was significantly increased [129]. Moreover, it was indicated that the TIBC was significantly elevated in mice exposed to Cd for 19 or 21 months. Although serum iron levels appeared unchanged after long-term Cd exposure, hepatic iron concentrations were significantly reduced [129]. Additionally, the UIBC and TIBC were markedly increased following prolonged Cd exposure [129]. These findings suggest that a compensatory biological response to iron deficiency may involve an increased amount of transferrin available for iron binding in the serum.

**Figure 3:**
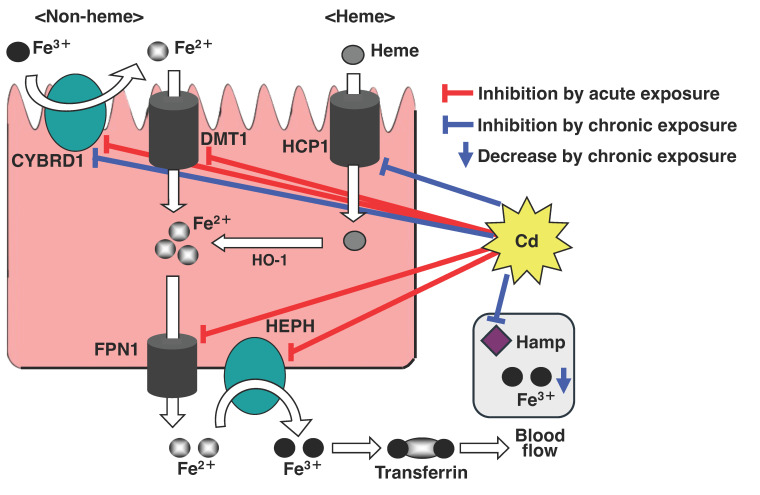
Scheme of the inhibitory effect of Cd on Fe transport. Cd inhibits iron absorption from duodenal epithelial cells by suppressing expressions of the iron transport-related genes, *DMT1*, *FPN1*, *DCYTB*, *HEPH*, and *HCP1*. In particular, the long-term exposure to Cd depletes hepatic iron levels and the expression of Hamp, a regulatory factor of iron homeostasis.

These results demonstrate that in mice, oral Cd intake rapidly downregulates the expressions of genes that encode duodenal iron transport-related molecules for non-heme and heme iron. On the other hand, in mice chronically exposed to Cd, iron absorption was inhibited and iron was released from the liver into the blood. Consequently, Cd exposure inhibits iron absorption from the gastrointestinal tract, and the resulting lack of iron in the body likely leads to iron-deficiency anemia (Figure 3).

## 6. Conclusions

**Figure 4:**
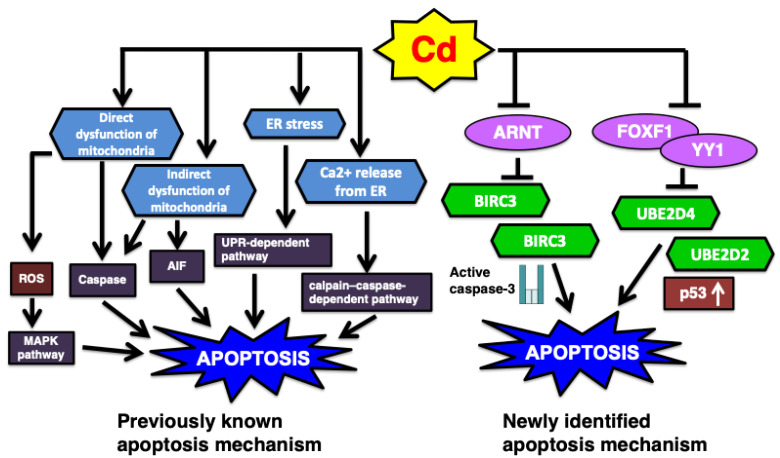
The summary of previous and newly identified Cd-induced apoptosis pathway. (1) Classically it was demonstrated that ER stress and mitochondria mediated apoptosis are involved in Cd renal toxicity. AIF, apoptosis-inducing factor. (2) Recent studies novelly identified the Cd-induced apoptosis pathways such as UBE2D-regulated p53 and ARNT-modified BIRC3 mechanisms.

Various toxic chemicals induce necrosis and apoptosis, which are the major mechanisms of cell death [96,130,131,132,133]. In particular, toxic heavy metals and metalloids, such as methylmercury, inorganic lead, and inorganic arsenic, induce apoptosis in their respective target tissues [96,130,131,132,134,135]. Apoptosis is a primary toxic mechanism induced by Cd exposure through various pathways [83,118,136,137]. Previous review has summarized the involvement of apoptosis pathway in the Cd-induced renal toxicity: (1) the endoplasmic reticulum (ER)-mediated pathway, which involves ER stress and calcium release, subsequently triggering apoptosis via the unfolded protein response (UPR)-dependent and calpain–caspase-dependent mechanisms; and (2) the mitochondria-mediated pathway, in which Cd directly or indirectly induces mitochondrial dysfunction, leading to apoptosis through both caspase-dependent and caspase-independent mechanisms [138]. In this review of our recent studies on Cd toxicity, the transcription factors YY1, FOXF1, and ARNT are introduced as newly proposed regulators of apoptosis (Figure 4).

In addition, the downstream factor of each transcription factor is summarized. Recent research proposes that regulating the intracellular ATP level is important for cell viability [139,140]. Cd-inhibited MEF2A transcription activity is involved in decreased ATP levels via inhibition of glucose transportation. Moreover, the PPARδ transcription factor is proposed to be a modification factor for Cd toxicity. Genetic polymorphisms in the *PPARD* gene have been reported [141,142], and alterations in PPARδ expression or activity may contribute to individual variability in susceptibility to Cd-induced renal toxicity.

Finally, we summarized the molecular mechanism of Cd-induced anemia in this review. Anemia remains a significant global public health concern [143,144,145]. Our findings indicate that chronic exposure to Cd may aggravate iron deficiency. Accordingly, it is recommended that individuals at high risk for anemia reduce dietary and lifestyle-related Cd exposure—particularly from sources such as certain foods and tobacco products—and consider iron supplementation as a preventive strategy against anemia and its associated health complications.

A recent study to identify transcription factors involved in Cd toxicity using the liver and kidney of mice exposed to Cd for 3 months showed Cd accumulation in the liver (63.41 ± 5.80 µg/g) and kidney (97.56 ± 9.48 µg/g); however, only minimal hepatic toxicity was observed [146]. Another study reported that 12 months of Cd exposure resulted in a renal Cd concentration of 174.74 µg/g, and that this accumulation induced apoptosis in the mouse kidney [53]. Therefore, transcriptional changes in the kidneys of mice exposed to Cd for 3 months may contribute to the development of Cd-induced renal dysfunction. Research based on altered transcription factors is expected to elucidate the molecular mechanism involved in the early stages of Cd renal toxicity.

Thus, it was found that multiple target molecules, including transcription factors and their downstream genes, are strongly involved in the expression of Cd toxicity. Chronic renal toxicity of Cd induces cell death, such as apoptosis and necrosis, following dysfunction in proximal tubular cells. At these stages, Cd appears to affect multiple target molecules, such as those presented in this review. At present, only a part of the molecular mechanism of Cd toxicity has been elucidated, and the whole picture is not clear. Further research is needed to clarify the whole picture of the molecular mechanisms of Cd toxicity.

## Figures and Tables

**Table 1:** Maximum levels for Cd in selected food categories as established by the Codex Alimentarius Commission.

Commodity/ Product Name	ML (mg/kg)	Commodity/ Product NAME	ML (mg/kg)	Commodity/Product Name	ML (mg/kg)
Brassica vegetables	0.05	Stalk and stem vegetables	0.1	Natural mineral waters	0.003 *
Bulb vegetables	0.05	Cereal grains	0.1	Salt, food grade	0.5
Fruiting vegetables	0.05	Rice, polished	0.4	Chocolates containing or declaring <30% total cocoa solids on a dry matter basis	0.3
Leafy vegetables	0.2	Wheat	0.2	Chocolate containing or declaring ≥30% to <50% total cocoa solids on a dry matter basis	0.7
Legume vegetables	0.1	Quinoa	0.15	Chocolate containing or declaring ≥50% to <70% total cocoa solids on a dry matter basis	0.8
Pulses	0.1	Marine bivalve molluscs	2	Chocolate containing or declaring ≥70% total cocoa solids on a dry matter basis	0.9
Root and tuber vegetables	0.1	Cephalopods	2	Cocoa powder (100% total cocoa solids on a dry matter basis) ready for consumption	2.0

ML, maximum level; * the ML is expressed in mg/L.
